# Thy-1 plays a pathogenic role and is a potential biomarker for skin fibrosis in scleroderma

**DOI:** 10.1172/jci.insight.149426

**Published:** 2022-10-10

**Authors:** Roberta G. Marangoni, Poulami Datta, Ananta Paine, Stacey Duemmel, Marc Nuzzo, Laura Sherwood, John Varga, Christopher Ritchlin, Benjamin D. Korman

**Affiliations:** 1 Division of Allergy, Immunology and Rheumatology, Department of Medicine, University of Rochester Medical Center, Rochester, New York, USA.; 2 Division of Rheumatology, Department of Internal Medicine, University of Michigan, Ann Arbor, Michigan, USA.

**Keywords:** Dermatology, Immunology, Fibrosis, Skin

## Abstract

Thy-1 (CD90) is a well-known marker of fibroblasts implicated in organ fibrosis, but its contribution to skin fibrosis remains unknown. We examined Thy-1 expression in scleroderma skin and its potential role as a biomarker and pathogenic factor in animal models of skin fibrosis. Skin from patients with systemic sclerosis demonstrated markedly elevated Thy-1 expression compared with controls, colocalized with fibroblast activator protein in the deep dermis, and correlated with the severity of skin involvement (modified Rodnan skin score). Serial imaging of skin from Thy-1 yellow fluorescent protein reporter mice by IVIS showed an increase in Thy-1 expression that correlated with onset and progression of fibrosis. In contrast to lung fibrosis, Thy-1–KO mice had attenuated skin fibrosis in both bleomycin and tight skin-1 murine models. Moreover, Thy-1 regulated key pathogenic pathways involved in fibrosis, including inflammation, myofibroblast differentiation, apoptosis, and multiple additional canonical fibrotic pathways. Therefore, although Thy-1 deficiency leads to exacerbated lung fibrosis, in skin it is protective. Moreover, Thy-1 may serve as a longitudinal marker to assess skin fibrosis.

## Graphical abstract

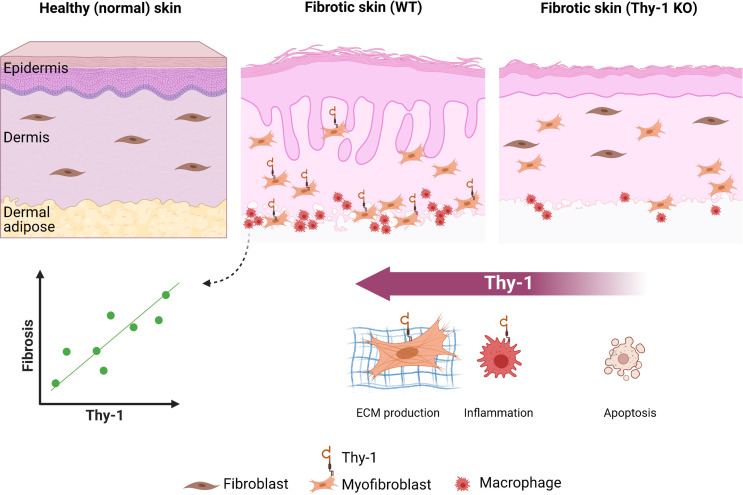

## Introduction

Systemic sclerosis (scleroderma, SSc) is a prototypical multisystem fibrotic disease characterized by excessive matrix production by activated fibroblasts (1). The etiology of skin fibrosis, a hallmark of SSc, is multifactorial and marked by a complex interaction of cells and cytokines that promote profibrotic signaling pathways and resultant mechanical forces (2).

Thy-1 (CD90), a glycosylphosphatidylinositol-anchored (GPI-anchored) glycoprotein highly expressed in neurons, T cells, and endothelial cells, has long been recognized as a fibroblast marker (3). Thy-1 has multiple physiologic functions, including cell-cell signaling, mechanotransduction, and cellular differentiation (4). In fibroblasts, Thy-1 specifically regulates cell adhesion and myofibroblast differentiation through integrin signaling and is directly involved in Fas-mediated apoptosis (5, 6). Thy-1 also regulates the PPARγ pathway and orchestrates mesenchymal cell differentiation between adipogenic and osteogenic fates (7, 8).

Lung fibroblast heterogeneity can be viewed on the basis of Thy-1 positivity (9), and likewise, synovial fibroblast subsets are defined anatomically and functionally by expression of Thy-1 and additional markers including cadherin-11, CD34, fibroblast activation protein (FAP), and MHC class II (10–12).

In skin, distinct fibroblast lineages are defined geographically based on their presence in the papillary or reticular dermis (13, 14). Of interest, these fibroblast subtypes have previously been defined based on their expression of Thy-1 and FAP with Thy-1–positive cells residing primarily in the reticular dermis (13).

While Thy-1 is a marker of fibroblast subsets, the specific actions of this molecule in the process of fibrosis are less established. In animal models of fibrosis, Thy-1 modulates fibrotic pathways in the lung, liver, and orbit (9, 15, 16). In the lung, loss of Thy-1 exacerbates and slows the resolution of fibrosis, and these effects are reversed by pharmacologic treatment with Thy-1 replacement (6, 17). In the skin, fibrosis has not been investigated, but Thy-1 deficiency or silencing leads to impaired wound healing (18, 19), suggesting that either skin fibroblasts are functionally distinct from those that reside in the lung or Thy-1 has different effects in wound healing and fibrosis.

Thy-1 is overexpressed in SSc skin and serum (20, 21), but the function of Thy-1 in SSc or other human skin diseases is unknown. In this study, we assessed Thy-1 as a marker of skin fibrosis and looked to elucidate its potential pathologic role in SSc.

## Results

### Thy-1 is increased in SSc skin fibroblasts and correlates with disease severity.

**Figure 1:**
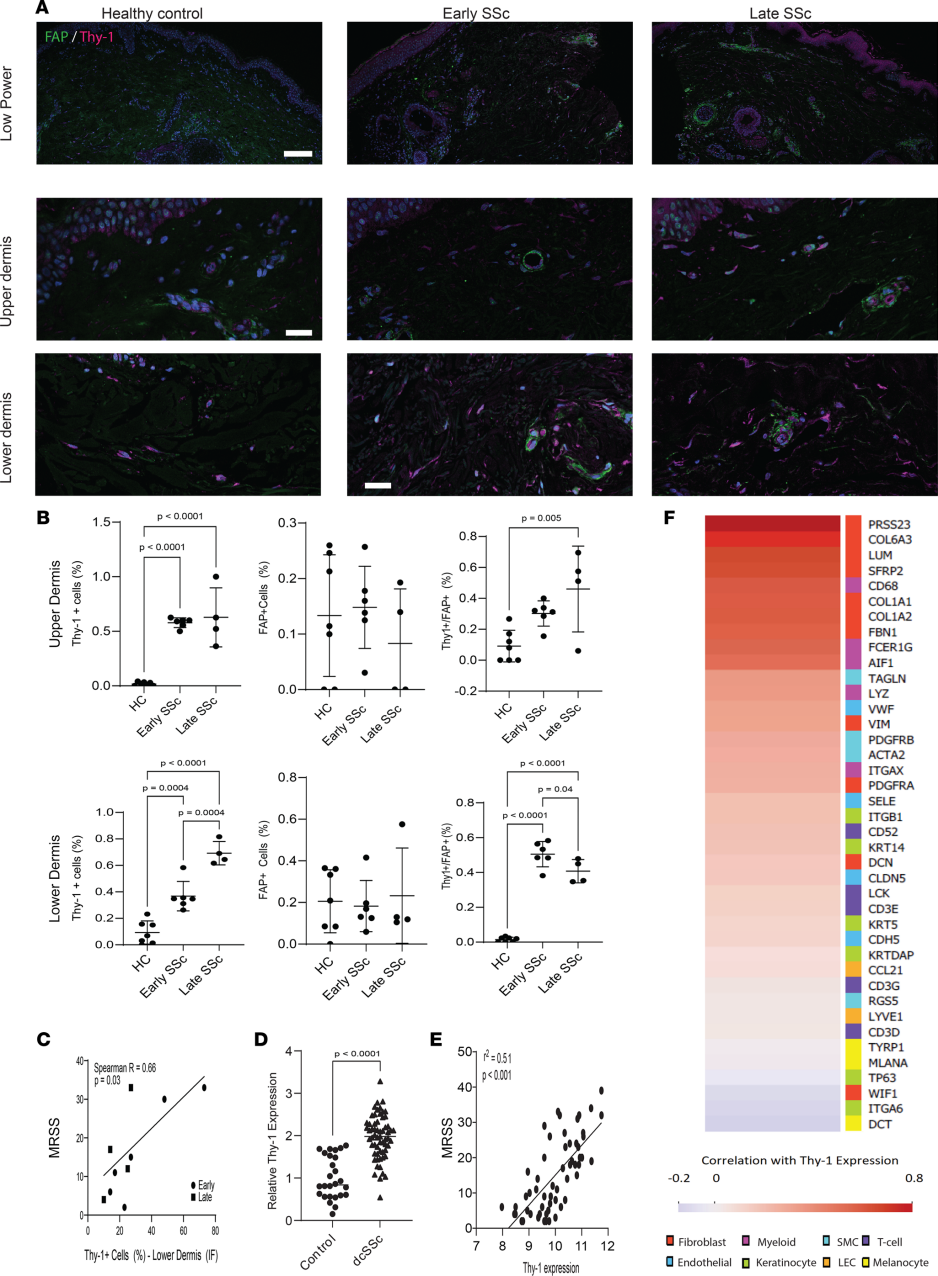
Increased Thy-1 expression in SSc skin biopsies. (**A**) Immunofluorescence using Abs to Thy-1 (magenta) and FAP (green); nuclei stained with Hoechst (blue). Skin biopsies from patients with diffuse SSc with early (< 3-year duration, *n* = 7), late (> 3-year duration, *n* = 4) disease and age-matched healthy controls (*n* = 6). Representative photomicrographs. Scale bars: 250 μm (upper row), 100 μm (lower 2 rows). (**B**) Quantification of cells within the upper (top panel) and lower dermis (bottom panel) immunopositive for FAP (left), Thy-1 (middle) per total cells in 3 high-power fields per participant, and FAP^+^Thy-1^+^ cells per total Thy-1^+^ cells (right) in 3 high-power fields per participant. ANOVA with Tukey post hoc test. Bars represent means with SDs. (**C**) Correlation between Thy-1^+^ cells in the reticular dermis with the MRSS. (**D**) Thy-1 gene expression was assessed in a SSc skin biopsy transcriptome data set (GEO GSE58095). *P* values calculated using 2-sided *t* test. *n* = 64 SSc and 38 controls. Data are presented as means ± SDs. (**E**) Correlation between Thy-1 gene expression and MRSS. Spearman’s rank correlation test. *n* = 63. (**F**) Heatmap representing correlation between Thy-1 expression and expression of genes demonstrated to identify subpopulations of skin cells. Scale represents correlation (Spearman’s *r*) and genes are listed in order from highest to lowest correlation with colors on the right corresponding to cellular populations marked by each gene. SMC, smooth muscle cells; LEC, lymphatic endothelial cells.

To investigate Thy-1 expression in SSc, we performed immunofluorescence staining in skin biopsies from healthy controls and patients with SSc (Supplemental Table 1; supplemental material available online with this article; https://doi.org/10.1172/jci.insight.149426DS1). Remarkably, while there was almost no expression of Thy-1 in the reticular dermis from healthy control skin (9.3% ± 8.7%), Thy-1 was expressed in SSc with increasing levels over time in early-stage patients (disease duration < 3 years) demonstrating moderate expression (36.8% ± 11.1%) and later-stage patients showing high expression (69.2% ± 8.9%) (Figure 1, A–C). It is known that FAP and Thy-1 expression status define subsets of fibroblasts in the skin, with FAP^+^ cells localizing primarily to the papillary dermis and Thy-1^+^ fibroblasts to the reticular dermis, with a small number of double-positive cells in the transition zone (13). Therefore, to further characterize SSc fibroblasts, we assessed coexpression of Thy-1 with FAP (Figure 1A and Supplemental Figure 1 for higher magnification) and observed a remarkable change to FAP^+^Thy-1^+^ cells in SSc (45.3% ± 6.9% Thy-1^+^ double-positive cells, compared with 1.4% ± 1.1% in controls) but did not note substantial differences in double-positive cells in early versus late SSc.

We next explored the gene expression of Thy-1 in skin from patients with SSc in 2 publicly available microarray data sets (NCBI Gene Expression Omnibus [GEO] GSE58095 and GSE76886). We observed a significant increase in expression of Thy-1 in SSc across cohorts and elevated levels in both limited and diffuse cutaneous SSc (Figure 1, B and D, and Supplemental Figure 2). We also found a striking positive correlation between Thy-1 expression and severity of skin fibrosis measured by the modified Rodnan skin score (MRSS) (Figure 1E; *r* = 0.63, *P* < 0.0001).

We next assessed Thy-1 lung expression in a publicly available data set (GEO GSE48149) and found that Thy-1 was significantly elevated in SSc lung explants (Supplemental Figure 3). This data set included patients with SSc with either interstitial lung disease (ILD) or pulmonary hypertension, patients with idiopathic pulmonary hypertension, and patients with idiopathic pulmonary fibrosis (IPF). Notably, all patients with SSc and with IPF had significantly elevated Thy-1 with a trend toward the largest increase in patients with SSc-ILD. A second smaller study (GEO GSE81292) did not show any significant difference in Thy-1 expression between SSc-ILD and controls.

To address whether Thy-1 expression was coming from fibroblasts or other skin cells, we correlated Thy-1 expression with genes specifically representing multiple skin cell types. We found strong correlations between Thy-1 expression and multiple fibroblast markers (PRSS23, SFRP2, FBN1, Col1a1, Col1a2, and LUM; Spearman *r* > 0.5) with no significant correlation in markers of most other cell types, including keratinocytes, smooth muscle cells, T cells, melanocytes, and endothelial cells (Figure 1F). A subset of myeloid genes (CD68 and FCER1) had strong correlation with Thy-1 while others (AIF1 and LYZ) demonstrated a moderate correlation.

### Thy-1 knockdown reduces fibrotic gene expression in SSc fibroblasts.

Dermal fibroblasts from healthy controls (*n* = 3) and SSc (*n* = 3) were explanted; consistent with gene expression and immunostaining, SSc fibroblasts demonstrated increased Thy-1 gene expression (3.38-fold increase compared with control, *P* = 0.1). After electroporation, cells were transfected with siNC or siThy-1 and treated with TGF-β (see Methods for full details). TGF-β–treated SSc fibroblasts demonstrated a significant reduction in TGF-β–induced α-SMA expression (*P* = 0.03) and showed a trend toward reduced Col1a1 expression, whereas no reduction in fibrotic gene expression was observed in healthy control fibroblasts (Supplemental Figure 4).

### Thy-1 can serve as a surrogate marker of in vivo skin fibrosis.

**Figure 2:**
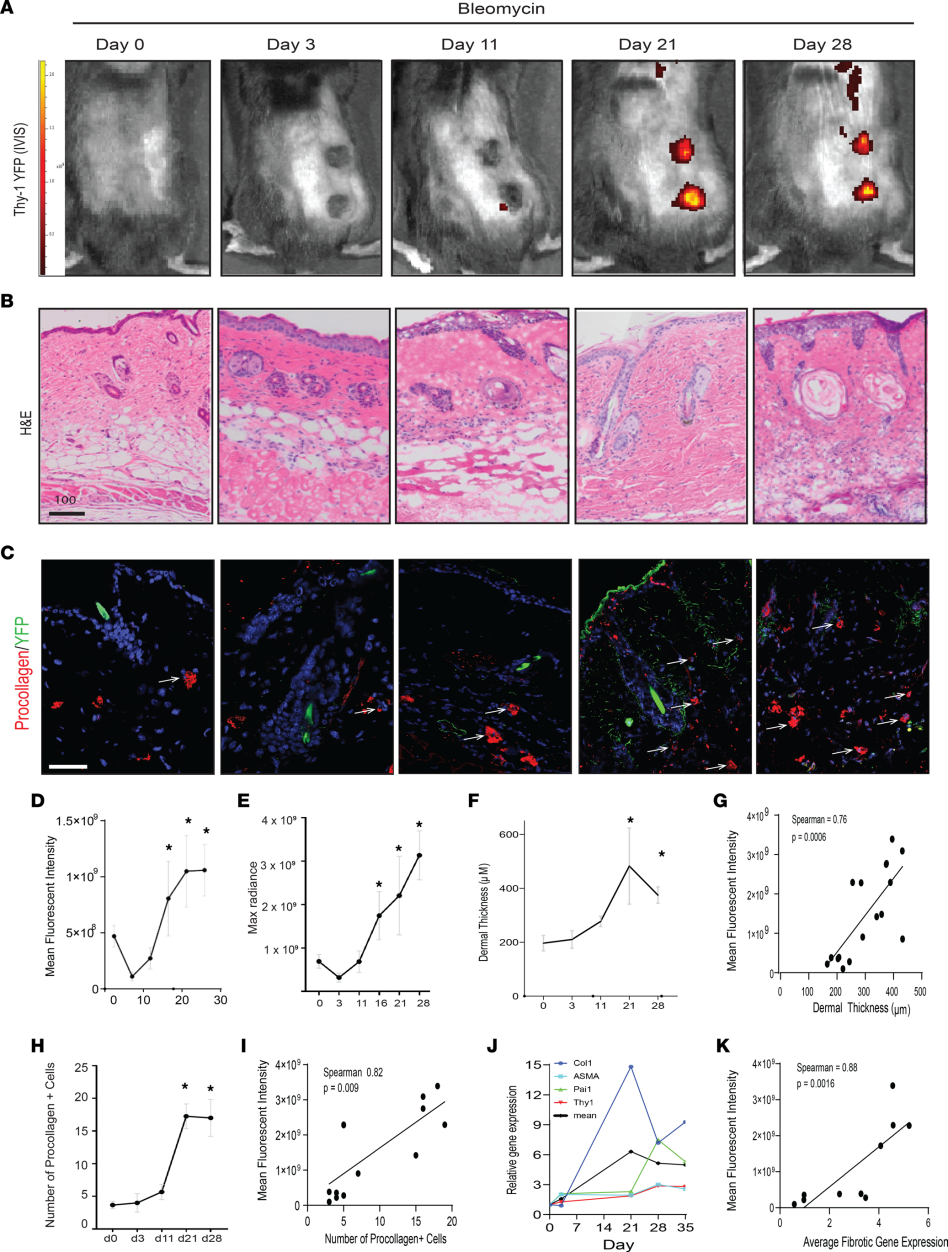
Thy-1 YFP can serve as an in vivo marker of skin fibrosis. (**A**) Representative images of YFP signal (radiance efficiency [photon/s/cm^2^/sr]/[μW/cm^2^]) measured at λ excitation max, 465 nm, and λ emission max, 520 nm by IVIS Spectrum after bleomycin injection. (**B**) Representative H&E-stained skin images showing temporal increase in dermal thickness induced by bleomycin. Scale bar: 100 μm. (**C**) Representative immunofluorescence imaging for Abs against pro–collagen type 1 (red, white arrowheads) as well as endogenous YFP label (green). Scale bar: 100 μm. (**D**) Average and (**E**) maximal YFP fluorescence radiance measured over time (days). Data are shown as mean ± SD. Tukey’s multiple-comparison test. *P < 0.05 compared with day 0. *n* = 3 per group. (**F**) Quantification of dermal thickness over time (days) in bleomycin-treated mice, as determined by 5 high-power fields per mouse. Values are the mean ± SD. *P* values calculated using 2-sided *t* test. *P ≤ 0.05 versus day 0. *n* = 3–4 per group. (**G**) Correlation between dermal thickness and YFP maximal fluorescence intensity by IVIS. Spearman’s rank correlation. (**H**) Number of procollagen-immunopositive cells per high-power field. Results are mean ± SD from 3 high-power fields per mouse. *P* values calculated using 2-sided *t* test. *P ≤ 0.05. *n* = 3. (**I**) Correlation between number of pro–collagen 1^+^ cells and YFP maximal fluorescence intensity. Spearman’s rank correlation test. (**J**) Gene expression changes over time (*PAI1*, ASMA, *COL1A1*, and *THY1*) in skin treated with bleomycin analyzed by qPCR. The thicker black line represents the mean of all fibrotic genes. Data represent mean gene expression per time point. *n* = 3–4. (**K**) Correlation between the average of fibrotic gene expression and YFP maximal fluorescence intensity. Spearman’s rank correlation test. *PAI1*, plasminogen activator inhibitor-1; ASMA, α–smooth muscle actin (*ACTA2*).

To interrogate whether Thy-1 can serve as a marker of fibrosis in vivo, we performed time course experiments in Thy-1 YFP reporter mice (22) treated with bleomycin and tracked expression of Thy-1 using YFP fluorescence intensity analyzed by IVIS imaging. Thy-1 expression increased over time in skin injected with bleomycin with maximal expression at days 21 and 28 (Figure 2, A, D, and E) at the time of maximal fibrosis (Figure 2, B, F, and J). Remarkably, Thy-1 fluorescence intensity strongly and positively correlated with multiple markers of fibrosis including dermal thickness (Spearman *r* = 0.76, *P* = 0.006; Figure 2, C and G), procollagen 1^+^ cells (Spearman *r* = 0.82, *P* = 0.009; Figure 2, H and I), and fibrogenic gene expression (Spearman *r* = 0.88, *P* = 0.0016; Figure 2K). These findings provide evidence that Thy-1 expression increases during fibrogenesis and can serve as a marker to noninvasively and longitudinally measure fibrosis in skin.

### Thy-1–deficient mice have attenuated skin fibrosis.

**Figure 3:**
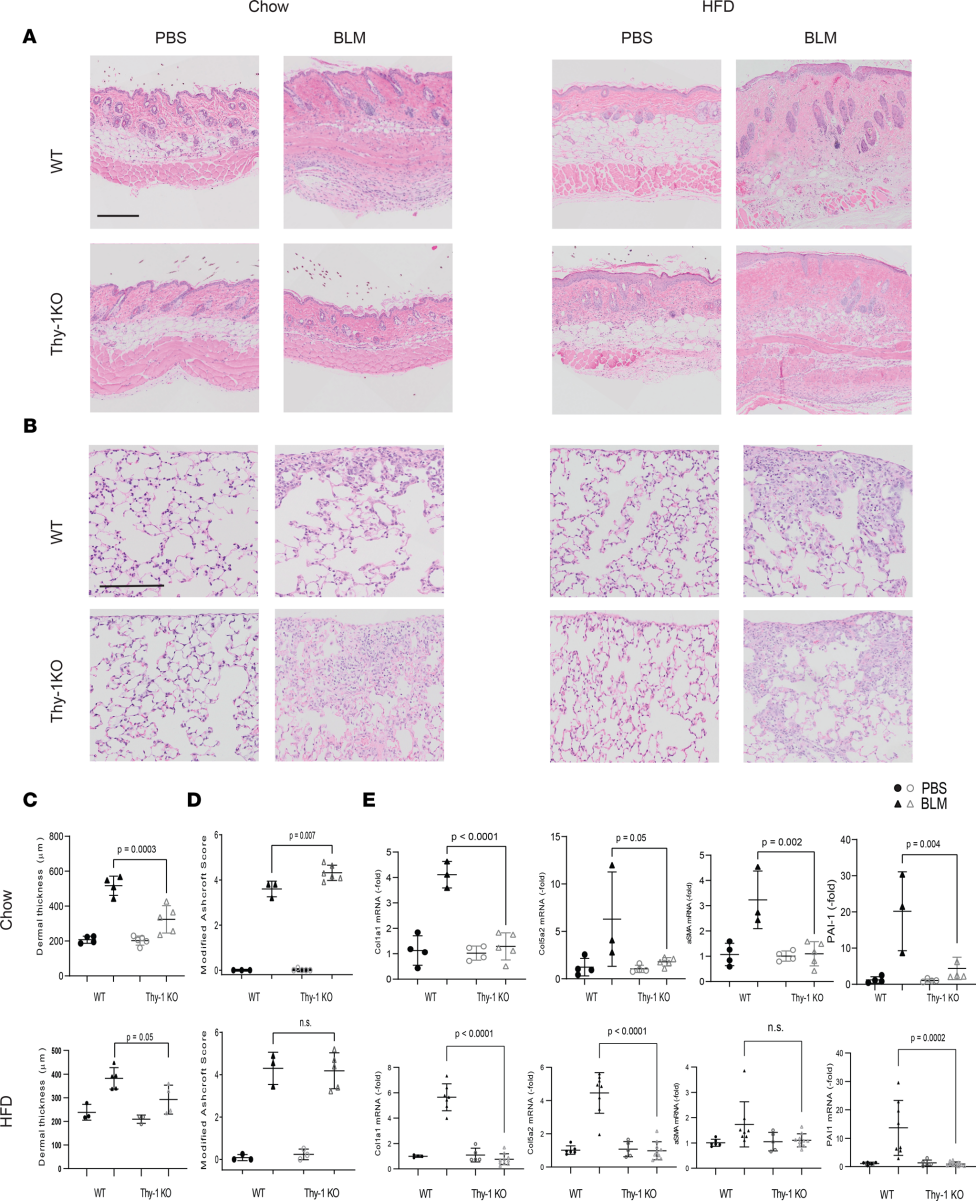
Thy-1–KO mice have attenuated bleomycin-induced skin fibrosis. (**A**) Representative images of H&E-stained skin after bleomycin or PBS injections in chow- and HFD-fed mice. Scale bar: 200 μm. (**B**) Representative H&E-stained lungs after bleomycin or PBS injections in chow and HFD-fed mice. Scale bar: 100 μm. (**C**) Dermal thickness as determined by 5 high-power fields per mouse (*n* = 3–5 mice/group). ANOVA with Tukey post hoc test. (**D**) Fibrosis scores (modified Ashcroft score). Results are mean ± SD from 10 high-power fields per mouse (*n* = 3–5 mice/group chow diet, *n* = 5–10 mice/group HFD). Mann-Whitney *U* test. (**E**) Expression of Col1a1, PAI-1, ASMA, and Col5a2 assessed by qPCR. Results were normalized to YWHAZ (*n* = 3–5 mice/group chow diet, *n* = 5–10 mice/group HFD). ANOVA with Tukey post hoc test.

To investigate the overall effect of Thy-1 in skin fibrosis, we performed in vivo experiments using mice deficient for Thy-1 (Thy-1 KO–mice) (23) in complementary models of fibrosis. Thy-1–KO mice that received s.c. injections of bleomycin demonstrated attenuation of dermal thickness compared with WT mice (Figure 3, A and C). Moreover, expression of fibrogenic genes including collagen (Col1a1 and Col5a2), ASMA, and PAI-1 measured by real-time PCR (qPCR) was substantially reduced in the Thy-1–KO mice (Figure 3B).

**Figure 4:**
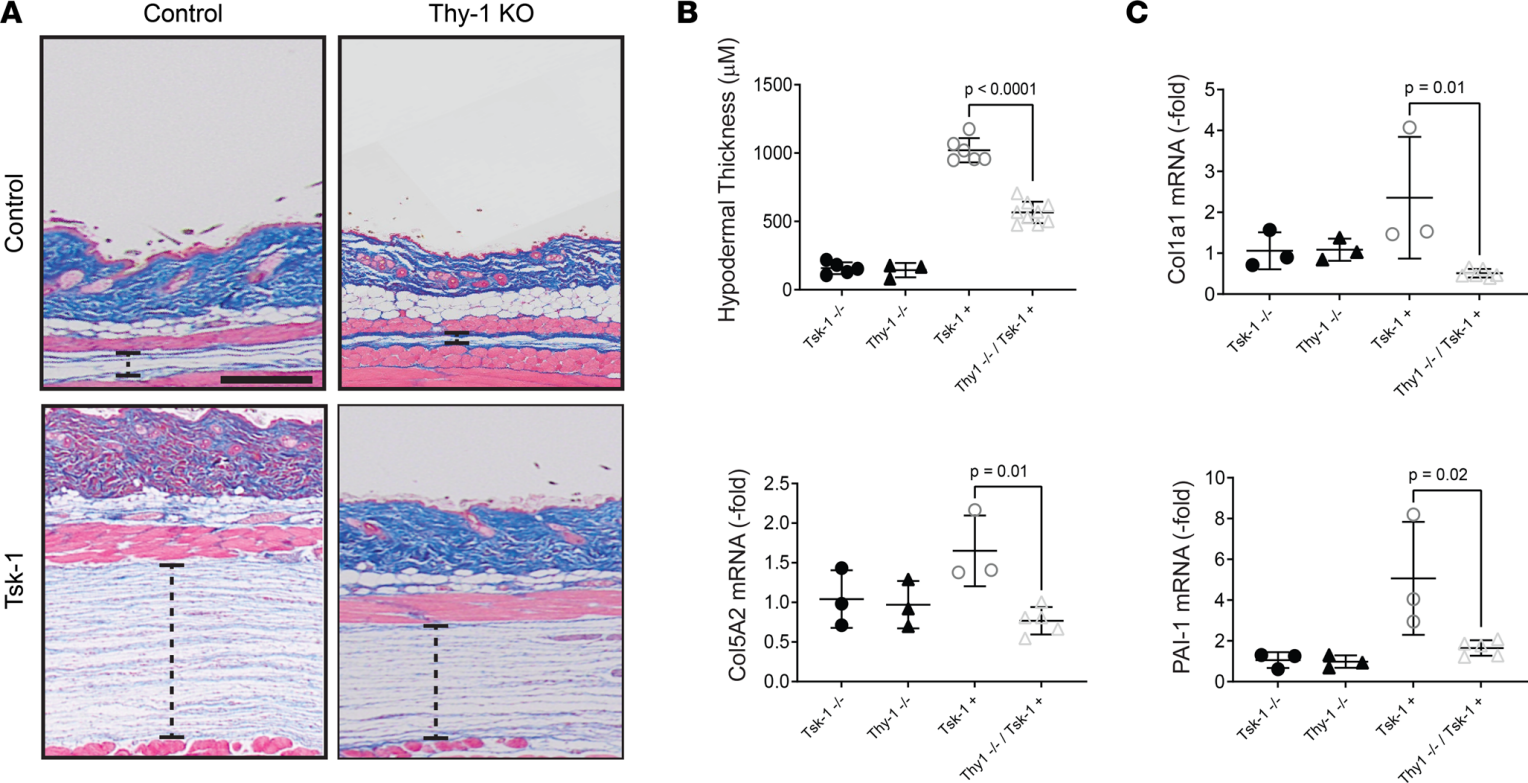
Tsk-1 mice lacking Thy-1 have attenuated skin fibrosis. (**A**) Representative Masson’s trichrome–stained images. Dashed line represents hypodermis. Scale bar: 250 μm. (**B**) Quantification of hypodermal thickness as determined by 5 high-power fields per mouse. *n* = 4–9 mice/group. ANOVA with Tukey post hoc test. (**C**) Expression of PAI-1, Col5a2, and Col1a1 assessed by qPCR. Results were normalized to YWHAZ (*n* = 3–6 mice/group). Results are mean ± SD. ANOVA with Tukey post hoc test.

To validate the effect of Thy-1 in skin fibrosis in a complementary genetic model, tight skin-1 (Tsk-1) mice (24) were crossed to Thy-1–KO mice (23). By 3 months of age, male Tsk-1 mice develop significant fibrosis of the hypodermis (25). Consistent with the bleomycin model, Tsk-1 mice deficient in Thy-1 were significantly protected from cutaneous fibrosis as measured both by hypodermal thickness and fibrotic gene expression (Figure 4, A–C).

Thy-1 has previously been shown to modulate adipogenesis (26), and Thy-1–KO mice fed a high-fat diet (HFD) develop increased obesity and bone loss (8, 27, 28). To assess whether an HFD would lead to an augmented skin fibrosis phenotype, Thy-1 KO were fed an HFD for 3 months and then treated with bleomycin. We found that in mice fed an HFD, Thy-1–KO mice were protected from skin fibrosis but to the same extent as mice fed a chow diet (Figure 3, D and E). This suggests that, unlike bone, where HFD plays a large role in defining mesenchymal cell fate (28), in skin fibrosis, other pathways are likely responsible for the protective effect of Thy-1 deficiency.

In direct contrast to the skin findings, Thy-1 deficiency has previously been shown to worsen lung fibrosis induced by intratracheal bleomycin (29). We confirmed these opposing findings in our s.c. bleomycin model: while Thy-1–KO mice had attenuated skin fibrosis, they demonstrated exacerbated lung fibrosis under chow conditions (Figure 3, D and E, and Supplemental Figure 5).

### Thy-1 regulates inflammation, myofibroblast differentiation, and apoptosis in skin fibrosis.

**Figure 5:**
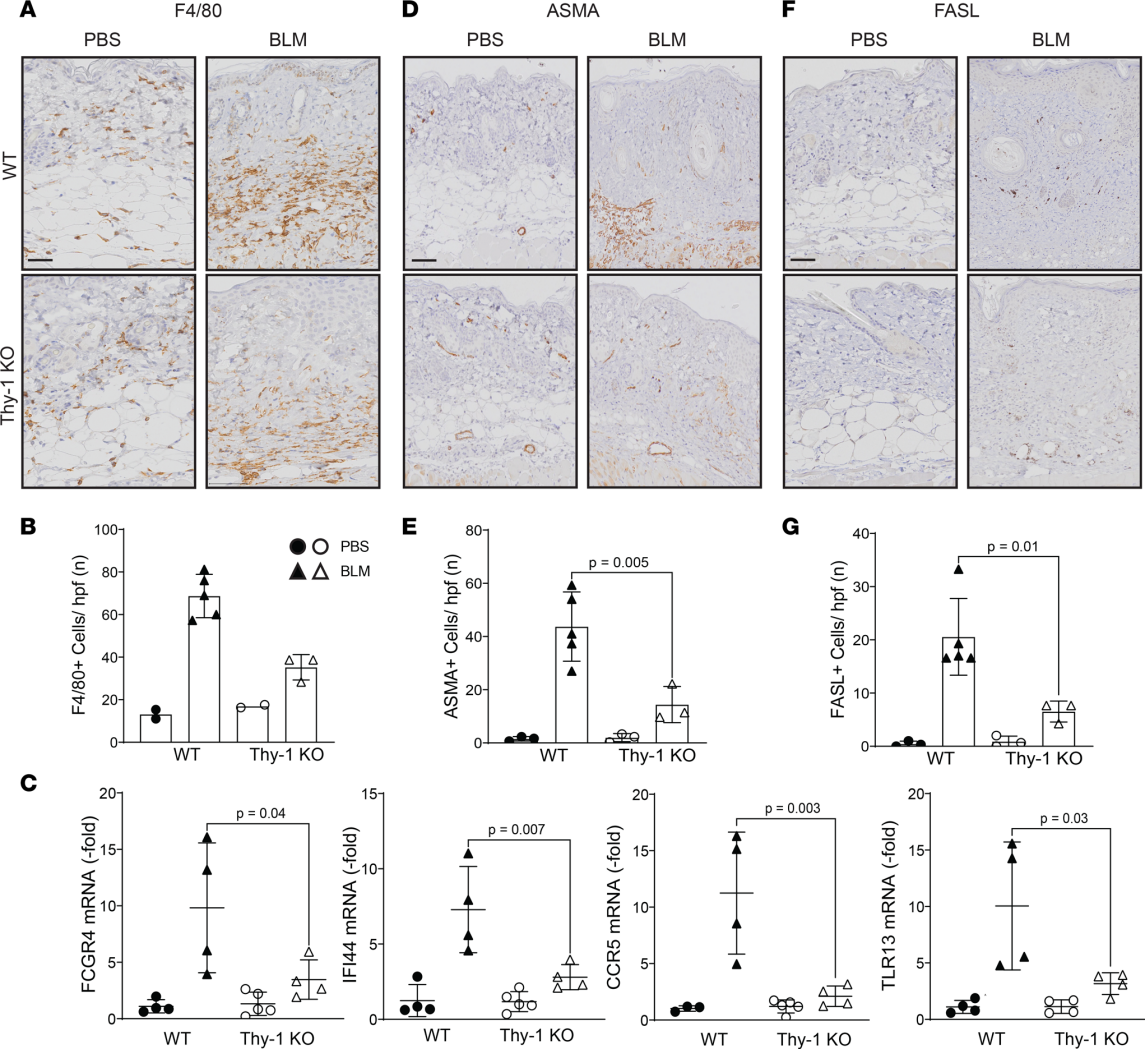
Thy-1–KO mice display decreased inflammation, number of myofibroblasts, and apoptosis during skin fibrosis. Representative IHC stains against F4/80 (**A**), ASMA (**D**), and FASL (**F**). Scale bar: 50 μm. Quantitative analysis of positive cells for F4/80 (**B**), ASMA (**E**), and FASL (**G**). Values are the mean ± SD of 3 high-power fields per mouse. ANOVA with Tukey post hoc test in **E**. *n* = 2–5 mice/group; solid circles represent WT mice and open circles represent Thy-1–KO mice. (**C**) Expression of FCGR4, IFI44 CCR5, and TLR13 assessed by qPCR. Results were normalized to YWHAZ (*n* = 4–5 mice/group). Results are mean ± SD. ANOVA with Tukey post hoc test.

It is well established that bleomycin-induced skin fibrosis leads to a macrophage-predominant inflammatory infiltrate (30), and we assessed if Thy-1 has an effect on these cells. We performed IHC for F4/80 as a marker of macrophages and investigated the expression of multiple inflammatory genes that we identified as significantly upregulated in skin fibrosis by bleomycin (inflammatory genes with > 10-fold increase at both day 7 and day 21 in the GEO data set GSE132869). We observed a remarkable decrease in the F4/80^+^ cells in the absence of Thy-1 (*P* = 0.002; Figure 5, A and B) and a significant downregulation in the expression of inflammatory genes FCGR4, IFI44, CCR5, and TLR13 (Figure 5C), suggesting that Thy-1 regulates multiple different inflammatory pathways during fibrosis.

Bleomycin leads to myofibroblast differentiation, and Thy-1–deficient mice show impaired apoptosis of myofibroblasts leading to nonresolving lung fibrosis (17). To examine whether the protective actions of Thy-1 deficiency in skin fibrosis are related to decreased numbers of myofibroblasts and regulation of apoptosis, we performed IHC staining for ASMA and FASL. We observed a significant decrease in the number of myofibroblasts (*P* = 0.01; Figure 5, D and E) and fewer FASL^+^ cells (*P* = 0.02; Figure 5, F and G) in the skin in the bleomycin-treated Thy-1–KO mice compared with WT.

These findings support the concept that Thy-1 is engaged and functional in multiple established mechanisms of skin fibrosis including regulation of inflammation, differentiation of myofibroblasts, and regulation of apoptosis.

### Thy-1 differentially modulates key fibrotic pathways.

**Figure 6:**
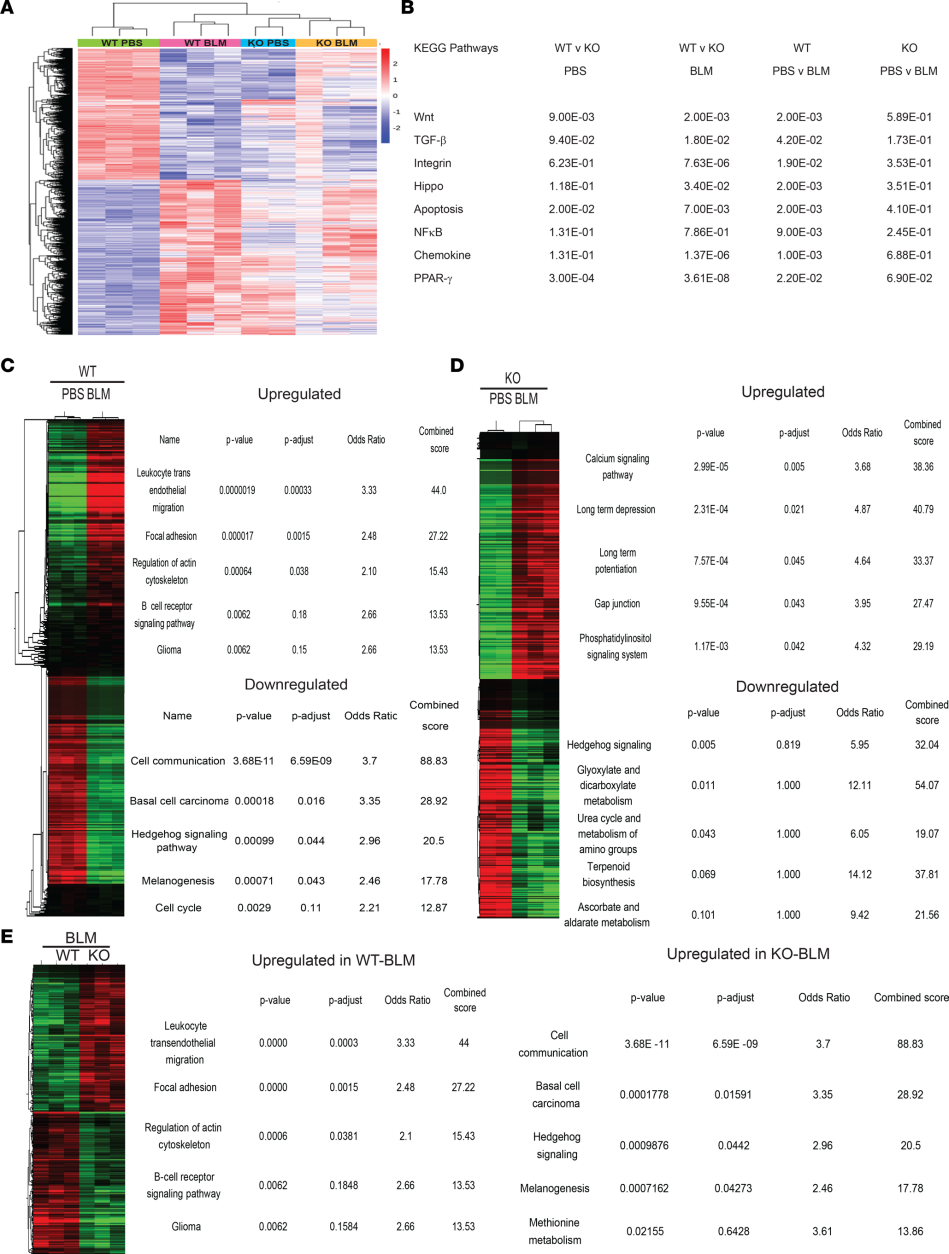
Thy-1 differentially modulates fibrotic pathways. (**A**) Heatmap of differentially expressed genes visualized using hierarchical clustering. *n* = 2–3 per group. (**B**) Pairwise pathway analysis for KEGG pathways implicated in fibrosis. Data represent *P* values for Tukey’s multiple-comparison test. (**C**) Differentially expressed KEGG pathways identified by Enrichr comparing PBS- and BLM-treated WT mice, (**D**) PBS- and BLM-treated Thy-1–KO mice, and (**E**) BLM-treated WT and Thy-1 KO. BLM, bleomycin.

Based on the findings that Thy-1’s effects are multifactorial, we performed bulk RNA-Seq on skin tissue from PBS- and bleomycin-treated WT and Thy-1–KO mice harvested at day 21 to identify the molecular mechanisms that underlie the antifibrotic effects. Our findings show a clear distinction between WT and KO mice from a global expression standpoint, and while there were marked changes between WT PBS- and bleomycin-treated mice, KO PBS- and bleomycin-treated mice had many fewer differentially expressed genes consistent with the ameliorated fibrotic phenotype (Figure 6A). Thy-1–KO skin also displayed a number of baseline changes compared with WT skin (Supplemental Figure 6). Examination of Kyoto Encyclopedia of Genes and Genomes (KEGG) pathways known to be relevant in fibrosis and regulation of Thy-1 revealed that TGF-β, Wnt, integrin, hippo (YAP/TAZ), PPARγ, apoptosis, NF-κB, and chemokine pathways were all significantly upregulated in WT bleomycin-treated mice but not in Thy-1–KO mice. All of these pathways except NF-κB were downregulated in Thy-1 KO bleomycin compared with WT bleomycin (Figure 6B). Unsupervised pathway analysis of WT bleomycin-treated mice showed overexpression of pathways consistent with fibrosis (i.e., focal adhesion) and inflammation (leukocyte transendothelial migration and TLR signaling) with downregulation of pathways related to skin cell homeostasis (cell cycle, basal cell carcinoma, and melanogenesis) (Figure 6C), while Thy-1–KO mice treated with bleomycin differentially expressed metabolic pathways (upregulation of calcium and phosphatidylinositol signaling, downregulation of urea cycle, glycoxylate, and terpenoid metabolism) (Figure 6D). Strikingly, when we compared the bleomycin-treated mice across genotypes (WT vs. Thy-1 KO), differential regulation of the same pathways seen in the WT PBS versus WT bleomycin comparison (overexpressed in WT compared with Thy-1 KO) was observed, suggesting that the Thy-1–KO mice are protected from the fibrotic and inflammatory pathways induced by bleomycin (Figure 6E).

## Discussion

In this study, we demonstrate that Thy-1 is aberrantly expressed in SSc fibroblasts and correlates with extent of fibrosis. We then demonstrate that Thy-1 is an in vivo marker of skin fibrosis and that loss of Thy-1 is protective in 2 complementary models of skin fibrosis. Thy-1 regulates a variety of relevant pathways including inflammation, myofibroblast activation, and apoptosis. Taken together, these findings indicate that Thy-1 serves as a marker of fibrosis and is involved in pathways that lead to skin fibrogenesis.

Thy-1 has long been considered a fibroblast marker (31). Thy-1 expression status can differentiate subtypes of fibroblasts, and in lung Thy-1^+^ and Thy-1^-^ fibroblasts are functionally distinct based on their ability to differentiate into myofibroblasts or lipofibroblasts (9). More recently, fibroblast subsets defined by Thy-1 have been further refined, particularly in the synovium where Thy-1 status along with the markers CD34 and cadherin 11 define lining and sublining synovial fibroblasts in human rheumatoid arthritis (11), while Thy-1 and FAP mark subsets of inflammatory and destructive phenotypes in murine arthritis (10).

The role of Thy-1 has been studied in lung fibrosis where it has been reported that Thy-1 plays an important role in lung fibrogenesis in IPF in which patients have been reported to have decreased Thy-1 expression (29). Moreover, treatment with soluble Thy-1 has shown to reduce bleomycin-induced lung fibrosis severity (6). No specific report has been made in SSc-ILD, and we therefore assessed Thy-1 expression in publicly available data sets and found that patients with SSc-ILD had significantly elevated Thy-1 expression in lung explants. Moreover, in a recent paper assessing SSc-ILD by single cell RNA-Seq, it appears that Thy-1 represents a general marker of fibroblasts in SSc-ILD rather than marking a specific subpopulation of fibroblasts (32) as has been suggested previously (33).

However, an opposite role has been reported for Thy-1 in cutaneous wound healing in which Thy-1 suppresses fibroblast proliferation and promotes apoptosis via β3 integrin signaling (18). In this context, blocking Thy-1 in wound beds reduces repair and hinders reepithelialization (19). Given these opposing findings, it is of particular interest that we demonstrated opposite effects in skin and lung fibrosis in Thy-1–KO mice.

As our skin findings were divergent from those reported in lung fibrosis (29), we confirmed that Thy-1–KO mice had increased lung fibrosis, suggesting that loss of Thy-1 truly has organ-specific effects that are profibrotic in lung and antifibrotic in skin. This may be related to Thy-1’s ability to regulation differentiation into myofibroblasts (6) in different tissue contexts and that global Thy-1 KO likely has pleiotropic and tissue context-specific effects. Creation of a cell-specific Thy-1–KO mouse would enable better characterization of the specific cells that may be driving these organ-specific effects.

In skin, Thy-1 does not mark a single fibroblast subpopulation and does not uniformly differentiate pathologic subtypes of fibroblasts because it also marks other mesenchymal cells (34). However, in recent studies, Thy-1 and FAP have been shown to delineate anatomically distinct subsets of fibroblasts with FAP^+^ cells primarily restricted to the papillary dermis and Thy-1^+^ cells to the reticular dermis (13). Moreover, similar to the lipofibroblast phenotype in lung, Thy-1^+^ but not FAP^+^ Thy-1^-^ fibroblasts are capable of adipogenic differentiation (13). In our studies of human skin, we noted a remarkable transition from predominantly FAP^+^Thy-1^–^ cells in healthy controls to FAP^+^Thy-1^+^ cells in SSc. The FAP^+^Thy-1^+^ population is not well defined in human skin but, given these cells’ functional role as immune effector fibroblasts in the synovial sublining in mouse joints, they may serve as proinflammatory cells in SSc. Our correlational analysis of Thy-1 expression with fibroblast genes in public data set interestingly demonstrated the highest correlation with 2 genes that have recently been identified in a subset of fibroblasts (SFRP2^hi^) that are uniquely overexpressed in SSc skin, specifically the subset marked by PRSS23 (35). Correlation analysis demonstrated that no skin cells other than fibroblasts and myeloid cells correlated with Thy-1. Whether myeloid cells are expressing Thy-1 or simply increased in tissues with increased Thy-1 is difficult to ascertain in this analysis, and further staining and/or single-cell sequencing studies should be done to clarify this point. Moreover, single-cell RNA-Seq may further refine SSc fibroblast populations defined by Thy-1, FAP, and other markers of interest.

Our finding that Thy-1 is upregulated in SSc skin and dermal fibroblasts validates previous reports (21, 36), and we identified an important correlation between Thy-1 expression and the MRSS. This correlation was strong (Spearman’s *r* = 0.63 using publicly available transcriptional data, *r* = 0.66 in immunostaining). The publicly available data did not have information available regarding disease duration, and while late biopsies had a higher percentage of Thy-1^+^ cell numbers than early biopsies (Figure 1B), regression analysis showed that disease duration was not associated with the number of Thy-1^+^ cells (*r* = 0.08, NS) and was not a significant covariate in the MRSS correlation.

Using Thy-1 YFP reporter mice, which were originally described as a tool to track neurons (22) and have been shown to mark inducible Thy-1 in cancer and wound healing (37), we found an aberrant expression of Thy-1 in skin injected with bleomycin in a manner that recapitulated the peak of fibrosis seen with histologic and gene expression parameters. These findings indicate that Thy-1 can longitudinally assess fibrosis in vivo. Taken together, this reporter mouse and the IVIS imaging system hold potential for inclusion in pharmacologic studies where noninvasive assessment of fibrosis could be evaluated over time.

While Thy-1’s role as a marker is of interest, its potential functional role is relevant to disease pathogenesis. Thy-1 deficiency in lung leads to worsening of fibrosis (6, 29), while in skin, Thy-1 deficiency impairs wound healing (18, 19). The major finding of our study is that lack of Thy-1 leads to attenuation of skin fibrosis in both bleomycin and Tsk models. To confirm the functional effect of this finding in patient samples, we performed siRNA knockdown experiments in healthy and SSc fibroblasts and found that knockdown of Thy-1 in SSc specifically ameliorated aSMA expression suggesting that Thy-1 may play a pathogenic role. However, due to limited power in the setting of a small sample size, further studies are needed to confirm these findings.

Thy-1 modulates adipogenesis in both skin and mesenchymal stem cells (26). Thy-1–KO mice are more susceptible to HFD-induced obesity (27, 28) and bone loss (8, 28). As our group has shown that adipose tissue plays a critical role in skin fibrosis (38–41), we investigated whether an HFD may be modulating the antifibrotic effect seen in Thy-1–KO mice. Interestingly, while we continued to see a protective effect in the Thy-1–KO mice, a substantial change in the phenotype was not observed under HFD conditions. This finding suggests that metabolism and adipose tissue are not the key pathways by which Thy-1 regulates skin fibrosis. The interpretation of these findings, however, is challenging because it is possible that proinflammatory effects of the HFD (42) and lipolysis (43) may counterbalance some of the antifibrotic effects or that loss of Thy-1’s protective role prevents significant further protection in this setting. These effects should be further explored using adipose-specific KO mice, different HFDs with less proinflammatory properties, or pharmacologic modulation of fatty acid pathways.

Thy-1 is known to have pleiotropic effects including regulating pathways known to modulate fibrosis such as integrin-signaling, inflammation, myofibroblast differentiation, apoptosis, and mechanotransduction (4, 26). We found that Thy-1 deficiency in bleomycin-treated skin involves regulation of multiple pathways. Thy-1–KO mice had decreased macrophage-driven inflammation and downregulation of proinflammatory genes representing TLR, IFN, B cell, and macrophage pathways. Consistent with the antifibrotic phenotype and the reports from wound healing (18), we also found reduced numbers of myofibroblasts. Moreover, we observed reduced FASL in skin from bleomycin-treated Thy-1–KO mice. Since lung fibrosis studies have shown that Thy-1 deficiency prevents resolution of fibrosis by regulating myofibroblast apoptosis (6), this raises the possibility that Thy-1 may play different functions in early and late stages of fibrosis.

To better characterize the multiple effects of Thy-1 in skin fibrosis, RNA-Seq was performed and revealed that multiple relevant pathways in skin fibrosis are regulated by Thy-1. Gene expression clearly differentiated bleomycin-treated Thy-1–KO mice from WT, and the pathways that were most differentially expressed are those critical for fibrosis including those linked to TGF-β, Wnt, focal adhesion, integrin, and PPARγ signaling. Further studies are needed to define the cell-specific mechanism and cell-specific KO mice or single cell RNA-Seq may clarify which cells are driving pathology.

In summary, we find that Thy-1 is overexpressed in SSc skin fibroblasts and correlates with severity of disease and that deficiency of Thy-1 is antifibrotic in skin. The primary protective mechanism appears to be driven by decreased inflammation, myofibroblast differentiation, and apoptosis. It is intriguing that the effect of loss of Thy-1 in skin is opposite to that seen in lung, and we were able to confirm that the effects are opposite in our systemic bleomycin model that confers fibrosis of both organs. These findings add both clarity and complexity to the actions exerted by Thy-1 in fibroblast, fibrosis, and skin biology. Given the increasingly important role of Thy-1 in defining functional subsets of fibroblasts across tissues, further elucidation of subsets of fibroblasts in SSc marked by Thy-1, FAP, and additional markers hold great promise to identify pathologic subsets of SSc fibroblasts.

## Methods

### Human skin tissue.

Paraffin-embedded skin biopsy sections were examined from 5 patients with SSc and 3 healthy controls obtained from the scleroderma registry at the University of Rochester Medical Center or Northwestern University. All patients with SSc fulfilled American College of Rheumatology criteria (44).

### Animals.

Thy-1–KO female mice (8 to 12 weeks old) (23), Thy-1 YFP mice (22) (catalog 003709, The Jackson Laboratory), both in the isogenic C57BL/6 background, and C57BL/6J (WT) (The Jackson Laboratory) were housed at constant temperature on a 12-hour light/12-hour dark cycle and given regular chow or HFD and water ad libitum. The HFD contained 60% fat (catalog D12492, Research Diets), and the mice were fed for 3 months. All mice were genotyped using genomic DNA isolated from tail biopsies using real-time PCR (Transnetyx). All control mice represent littermate controls. Both Thy-1–KO and Tsk-1 strains were backcrossed at least 10 generations with C57BL/6 before crosses were performed. The Tsk phenotype was assessed in Tsk-1 mice and double-transgenic mice by scruffing the neck as previously described (25), and the Tsk-1 single-transgenic mice uniformly demonstrated the Tsk phenotype.

### Bleomycin-induced fibrosis.

Mice were treated with bleomycin (1 mg/mL in PBS; 10 mg/kg/d) or PBS by daily s.c. injections in 2 spots on the dorsal back skin for up to 14 days and harvested at various time points as indicated. At the end of experiments, blood and tissues (skin and lung) were collected and processed for analysis. Each experimental group consisted of 3–5 mice, and experiments were repeated at least 2 times with consistent results.

### Tsk-1 model of fibrosis.

Male Tsk1^+^ mice (24) (catalog 014632, Jackson Laboratories) were crossed with female Thy-1–KO mice to generate Thy-1–deficient Tsk1^+^ progeny. Male mice were aged to 3 months, and full-thickness skin was harvested and processed for histology (Masson’s trichrome staining) and qPCR.

### Evaluation of fibrosis.

Harvested skin and lungs were fixed in 10% formalin, then embedded in paraffin, and 4 μm thick sections were stained with H&E or Masson’s trichrome. Thickness of the dermis was determined at 5 randomly selected locations/slide using QuPath (45).

### Lung fibrosis scores.

H&E-stained lungs were assessed for histological features consistent for fibrosis using the modified Ashcroft score as previously described (46). Lung fibrosis scoring was performed in a blinded fashion in 10 high-power fields (images at original magnification, ×20), which included 8 subpleural and 2 central lung regions.

### qPCR.

Upon harvest, tissues (skin and lung) were immersed in RNA*later* (QIAGEN) and stored at −80°C. The samples were homogenized with the Bullet Blender 24 Gold (Next Advance). RNA was isolated using the RNeasy Micro Kit or RNeasy Fibrous Mini Kit (QIAGEN), and cDNA was synthesized using the High Capacity cDNA Reverse Transcription Kit (Applied Biosystems, Thermo Fisher Scientific). qPCR was performed using SYBR GreenER qPCR SuperMix (Invitrogen, Thermo Fisher Scientific), and quantification of gene expression was performed as previously described (47). All samples were normalized to YWHAZ gene expression, and results are expressed as the fold change of C_t_ values (mean of 3 replicates) compared with controls, using the 2^-ΔΔCt^ formula.

### Immunofluorescence.

We incubated 4 μm thick, paraffin-embedded sections of mouse lesional skin with rat anti–procollagen I (1:100; clone M-58, MilliporeSigma) primary Abs, followed by species-appropriate secondary Abs conjugated to Alexa Fluor 594 (Invitrogen, Thermo Fisher Scientific, A21201). Nuclei were detected using DAPI. Healthy controls and SSc skin sections were incubated with sheep anti-FAP (1:100 dilution; catalog AF3715, R&D Systems, Bio-Techne) and rabbit anti–Thy-1 (1:50 dilution; catalog ab92574, Abcam) primary Abs, followed by species-appropriate secondary Abs conjugated to Alexa Fluor 488 (Invitrogen, Thermo Fisher Scientific, A21200) or 647 (Jackson Immunoresearch, 313-607-003). Nuclei were detected using Hoechst 33342 (NucBlue Live Ready Probes, Molecular Probes, Thermo Fisher Scientific). Slides were evaluated using a Nikon A1 laser scanning confocal microscope.

### IHC.

Then, 4 μm thick, paraffin-embedded sections of mouse lesional skin were processed for IHC using chromogenic enzyme substrate reactions with DAB (catalog K3468, DAKO, Agilent Technologies). Slides were deparaffinized on an automated platform (Leica Autostainer XL) and next treated with an antigen retrieval step using a sodium citrate solution at pH 6 in a pressure cooker. Primary Abs for FASL (1:500; clone ab5285, Abcam), F4/80 (1:750; Cell Signaling Technology), and ASMA (1:3,000; clone ab5285, Abcam) were incubated at 4°C overnight in a humid chamber. Secondary Ab incubation and chromogenic reactions were performed using an automated system (intelliPATH, Biocare), and specimens were counterstained with hematoxylin. The stained sections were digitized using a whole-slide imaging system (Olympus VS120), and quantitative analysis was performed using QuPath software(45).

### IVIS.

Thy-1 YFP mice (*n* = 18) were treated with PBS (*n* = 3) or bleomycin (*n* = 15) and imaged serially using fluorescence imaging on an IVIS Spectrum animal fluorescence scanner (PerkinElmer). Fluorescence images were acquired with YFP filter settings (λ excitation max, 465 nm, and λ emission max, 520 nm), and the minimum, mean, and maximum intensities (photons/s/cm^2^/sr) were quantified by Living Imaging Acquisition and Analysis software.

### Thy-1 skin expression in SSc.

To interrogate expression of Thy-1 in SSc, 2 publicly available microarray data sets (GEO GSE58095 and GSE76886) of skin biopsies from patients with SSc and healthy controls were assessed. Normalized expression of Thy-1 was first determined for each sample and compared across disease subsets and controls. Among SSc samples with available clinical data, Thy-1 expression was correlated with the MRSS, a validated measure of skin fibrosis (48). In lung tissue, Thy-1 expression in SSc and control lungs was assessed in 2 additional publicly available data sets (GEO GSE48149 and GSE81292)

### Thy1 gene knockdown in primary skin fibroblasts.

Primary skin fibroblast cells were generated from biopsied skin samples collected from healthy participants and patients with SSc. For Thy-1 gene knockdown, cultured primary skin fibroblast cells (passage 4) were harvested and transfected with siRNA targeting human Thy1 (Stealth RNAi siRNA targeting human Thy1, Assay HSS144275, Invitrogen, Thermo Fisher Scientific) or scrambled RNA (Stealth RNAi siRNA Negative Control, catalog 12935112, Invitrogen, Thermo Fisher Scientific) using a Neon transfection system (Invitrogen, Thermo Fisher Scientific). For this, 50 pmol siRNA was electroporated into skin fibroblast cells using the Neon transfection system in 10 μL tips at 1,400 mV/20 ms/2 pulse, and cells were replated in 24-well plates. A total of 18 hours after the transfection, cells were serum-starved for 8 hours and after that were cultured with or without 5 ng/mL of TGF-β1 for 24 hours (for RNA) or 48 hours (for protein). After that, cells were washed twice with PBS and harvested for RNA or protein isolation and subsequent quantification of mRNA and protein level expression levels of Thy1 and fibrosis-related genes.

### RNA-Seq.

RNA was extracted from skin of PBS- and bleomycin-treated WT and Thy-1–KO mice using RNeasy Micro Kit or RNeasy Fibrous Mini Kit (QIAGEN). Next, a genomic mRNA library was generated using a TruSeq stranded library (Illumina). RNA-Seq was performed using the NextSeq platform (Illumina) at a density of 20 million read pairs/library. DESeq2 (49) v1.22.1 within R 3.5.1 was used to perform data normalization and differential expression analysis with an adjusted *P* value threshold of 0.05 on each set of raw expression measures. The lfcShrink method was applied, which moderates log_2_ fold changes for lowly expressed genes. Significantly upregulated and downregulated genes (based on *P* adjusted < 0.05 and absolute [log_2_ fold change] > 0) were submitted to Enrichr (https://maayanlab.cloud/Enrichr/) to identify significantly enriched pathways dysregulated in each phenotype, and we assessed the top significantly enriched KEGG pathways based on pairwise comparison between upregulated or downregulated genes. RNA-Seq data have been deposited in the GEO database with accession number GSE211834.

### Statistics.

Results are presented as the mean ± SD unless otherwise indicated. A 1-way ANOVA with Tukey’s multiple-comparison test was used when more than 2 values were directly compared with each other. For 2-group comparisons, a 2-sided *t* test was used. Generally, experiments included at least 3 independent values from 2 independent experiments. *P* values less than 0.05 were considered statistically significant. In pairwise comparisons, Spearman’s rank method was used to assess correlation. Statistical analyses and graphs were performed with GraphPad Prism (GraphPad Software version 9).

### Study approval.

Animal studies complied with the Public Health Service Policy on Humane Care and Use of Laboratory Animals and all animal protocols were approved by the University of Rochester (number 102056). For human studies, samples were deidentified and were obtained as part of an IRB-approved protocol at the University of Rochester (study 1713). Written informed consent was obtained from all participants and was in accordance with the Helsinki Declaration.

Prior publication: This work was previously presented at the American College of Rheumatology Meetings November 8–13, 2019 (Atlanta, Georgia, USA), and November 5–9, 2020 (virtual), and the Keystone Symposia on Fibrosis February 19–23, 2020 (Victoria, British Columbia, Canada).

## Author contributions

RGM and BDK designed the study. RGM, PD, AP, SD, MN, LS, and BDK performed the experiments and analyzed the data. RNA-Seq analysis was performed by BDK. JV, AP, and CR gave conceptual advice and helped with the data interpretation and manuscript draft. RGM and BDK wrote the manuscript draft. All authors contributed to the draft review.

## Supplementary Material

Supplemental data

## Version 1 09/06/2022

In-Press Preview

## Version 2 10/10/2022

Electronic publication
