# Supplementary material for: Thy-1 plays a pathogenic role and is a potential biomarker for skin fibrosis in scleroderma
Source: JCI Insight. 2022 Oct 10;7(19):e149426. doi: 10.1172/jci.insight.149426 (PMC9675443; doi:10.1172/jci.insight.149426)
Supplement: Supplemental data [file jciinsight-7-149426-s097.pdf]

Table 1. Clinical characteristics of SSc patients and controls

Sex	Age	SSc/Control	Disease duration (yrs)	MRSS	Antibody (pattern)	Treatment
F	30	dcSSc	18	24	Scl-70	MMF
M	49	dcSSc	7	30	ANA (Nucleolar)	MMF
F	31	dcSSc	14	15	Scl-70	MMF
F	55	dcSSc	4	33	Scl-70	MMF
M	67	dcSSc	29	2	ANA (Nucleolar)	MMF
F	40	dcSSc	5	11	ANA (Nucleolar)	MMF
F	27	dcSSc	3	6	ANA (Nucleolar)	MMF
F	61	dcSSc	1	17	ANA (Speckled)	none
F	44	dcSSc	1	12	RNAP3	none
F	49	dcSSc	2	33	ANA (Speckled)	MMF
F	44	control				
F	42	control				
F	37	control				
F	26	control				
F	26	control				
M	23	control				
F	28	control				
F	57	control				

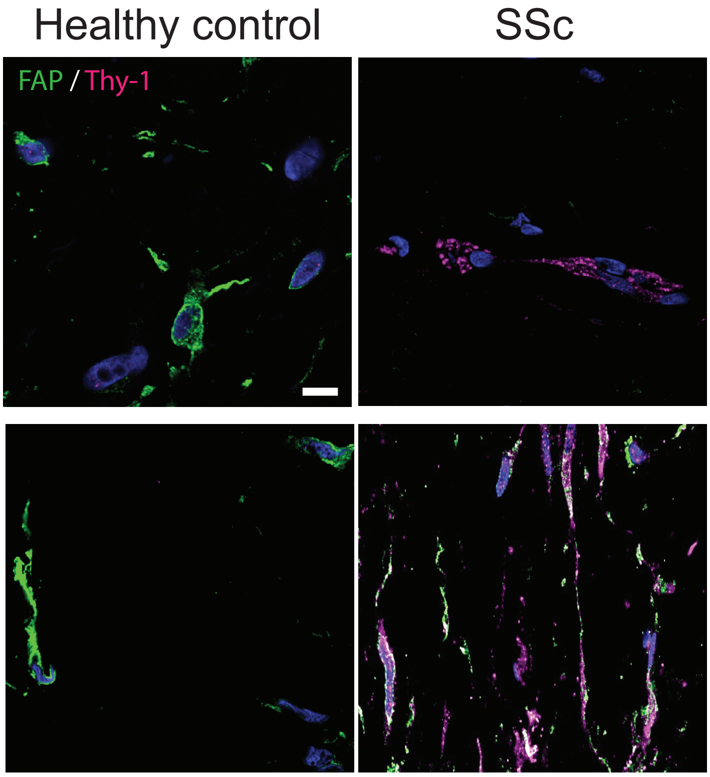

Supplemental figure 1. High magnification zoom images demonstrating Thy-1 and FAP co-localization

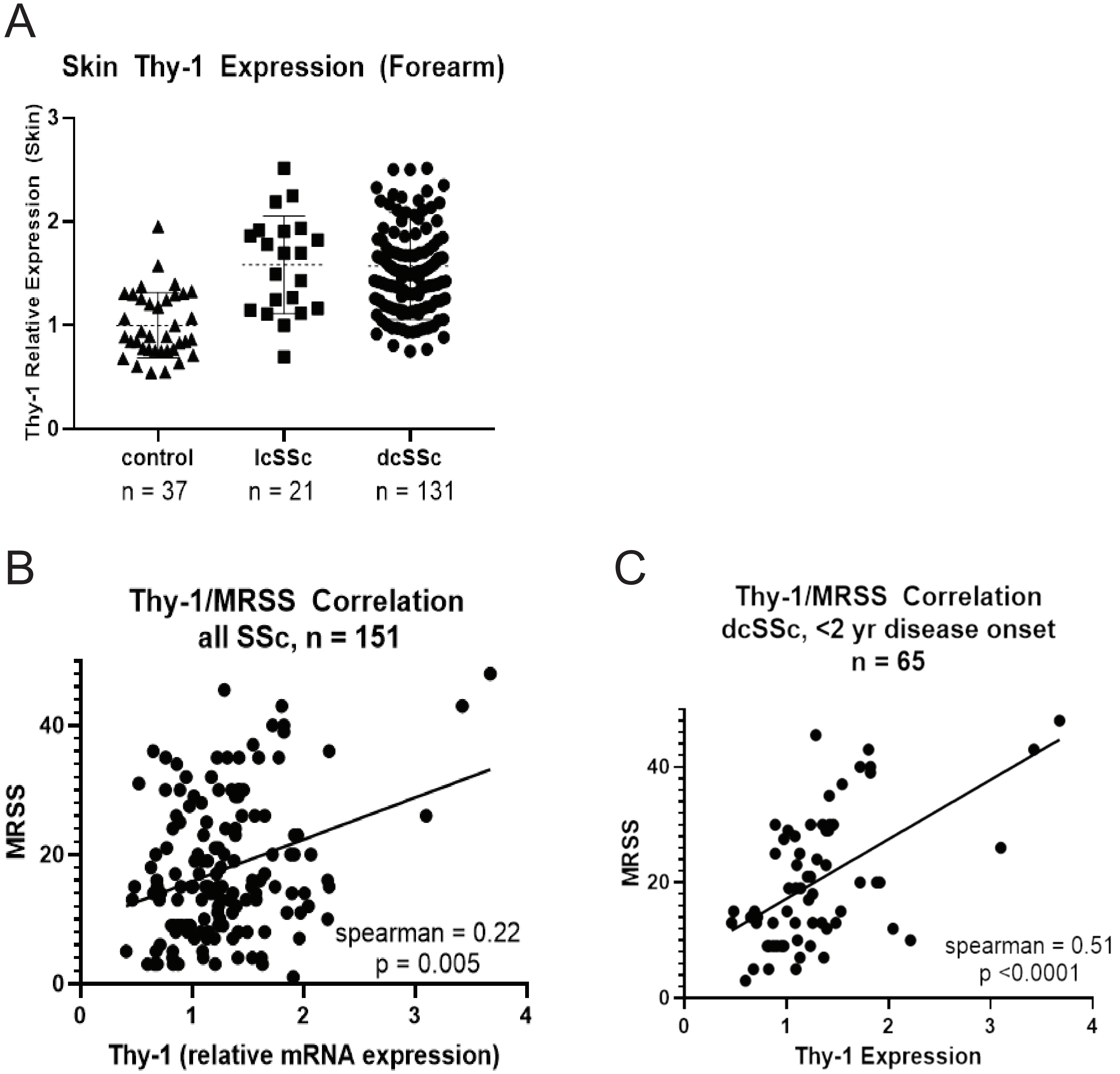

Supplemental Figure 2. Thy-1 expression is increased in SSc and correlates with skin fibrosis in an additional cohort. (A) Thy-1 gene expression was assessed in a SSc skin biopsy transcriptome dataset (GSE76886) and stratified by disease subtype. **(B)** Correlation between Thy-1 gene expression and modified Rodnan skin score (MRSS) amongst all SSc patients. Spearman's rank test. **(C)** Correlation between Thy-1 gene expression and MRSS amongst patients with diffuse cutaneous SSc and disease duration < 2 years. Spearman's rank test.

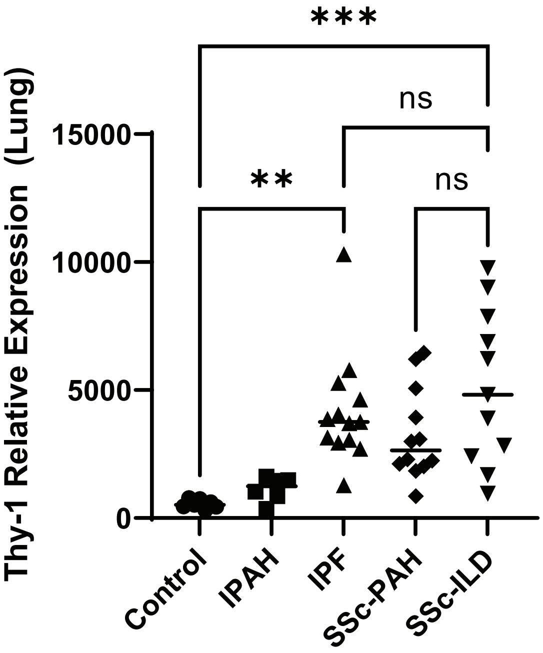

Supplemental Figure 3. Increased Thy-1 expression in SSc and IPF lung tissue.
Gene expression data from lung tissue from a previously performed microarray study (GSE48149) was queried for Thy-1 expression. Compared to control lung tissue, SSc patients (both SSc-PAH and SSc-ILD) demonstrated increased lung Thy-1. There was a similar increase seen in patients with idiopathic pulmonary fibrosis (IPF) but not idiopathic pulmonary hypertensino (IPAH).

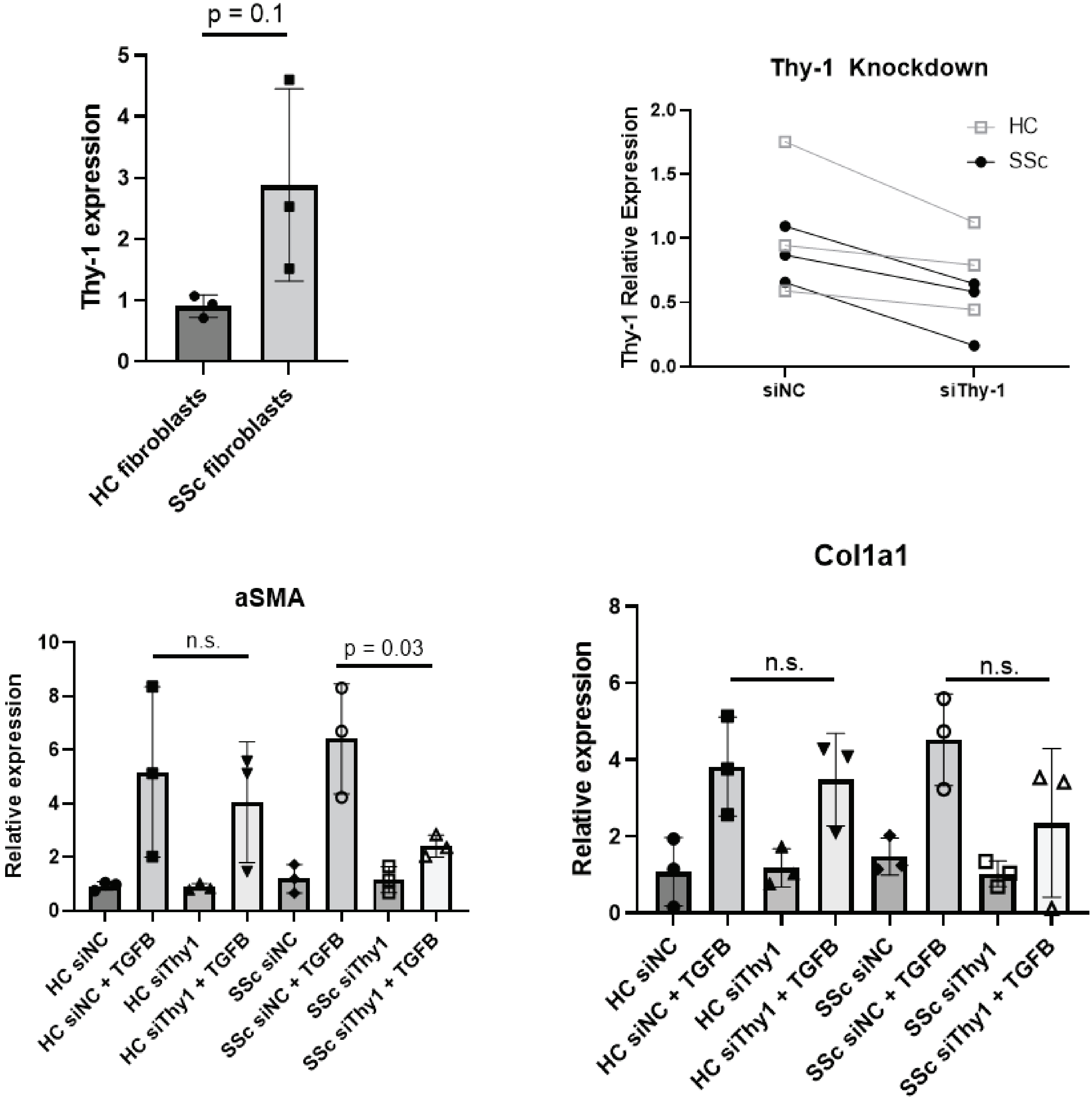

Supplemental Figure 4. Thy-1 knockdown ameliorate SSc fibroblast fibrotic gene expression.

Dermal fibroblasts were cultured from healthy donors (n=3) and patients with dcSSc (n=3). A. SSc fibroblasts had higher basal Thy-1 expression than control fibroblasts. B. Cells were electroporated and treated with siThy-1, qPCR for Thy-1 demonstrated 38 % (range 31-58%) knockdown. C. After treatment with either control siRNA or siThy1, cells were treated with TGF-b and fibrotic gene expression was assessed. SSc fibroblasts demonstrated a significant decrease in aSMA expression (p=0.03) and a non-significant trend toward decreased Cola1a1 expression while no decrease in these genes was seen in control fibroblasts.

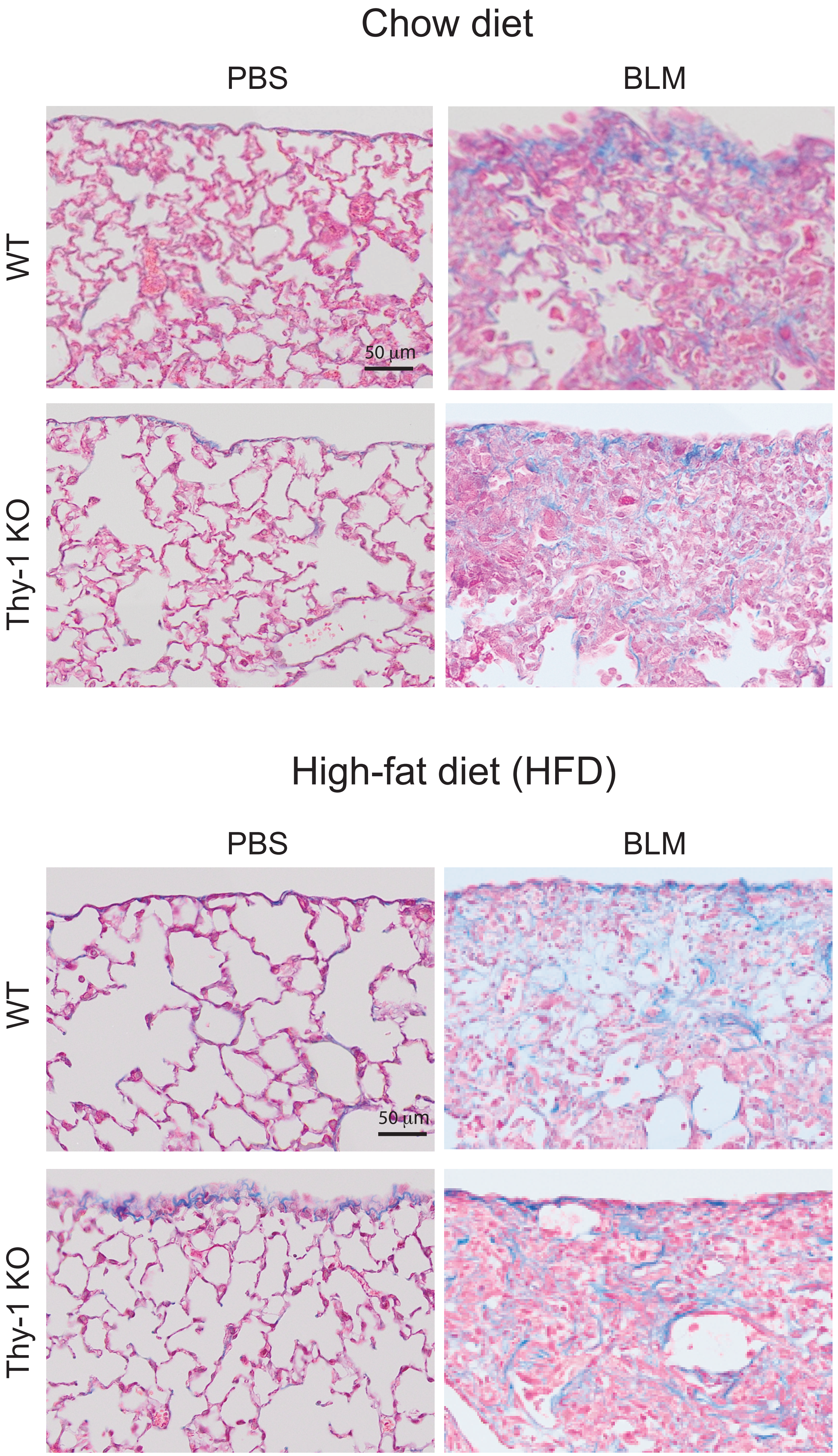

Supplemental Figure 5. Thy-1 deficiency leads to increased lung fibrosis induced by bleomycin. Female wildtype (WT) and Thy-1 KO mice were given daily subcutaneous injections of PBS or bleomycin (BLM) for 14 days. Lungs were harvested at day 21, processed for histology and stained with Masson's Trichrome, representative images. Scale bar: 50 µm.

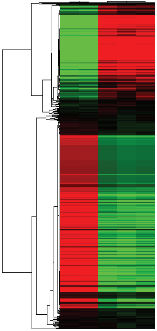

Upregulated

Name	P-value	Adjusted p-value	Odds Ratio	Combined score
Dilated cardiomyopathy (DCM)	8.296e-8	0.00002514	2.65	43.28
Aldosterone synthesis and secretion	2.001e-7	0.00002021	2.49	38.38
Toxoplasmosis	2.899e-7	0.00002196	2.42	36.42
cGMP-PKG signaling pathway	1.378e-7	0.00002088	2.13	33.60
Peroxisome	0.000002322	0.0001173	2.49	32.28

Downregulated

Name	P-value	Adjusted p-value	Odds Ratio	Combined score
RNA transport	9.044e-25	2.740e-22	2.91	160.93
Spliceosome	9.447e-23	1.431e-20	3.06	155.42
Proteasome	4.324e-12	4.368e-10	3.52	92.05
mRNA surveillance pathway	5.189e-11	3.144e-9	2.58	61.21
Ribosome biogenesis in eukaryotes	4.584e-11	3.472e-9	2.44	58.06

Supplemental Figure 6. Effect of Thy-1 deficiency in PBS-treated skin
Female wildtype (WT) and Thy-1 KO mice were given daily subcutaneous injections of PBS or bleomycin (BLM) for 14 days. Skin was harvested at day 21, RNA was isolated and processed for RNA-sequencing. Differential gene expression analysis was performed using deSeq2, and pathway analysis was performed using Enrichr. Differentially expressed pathways between comparison of PBS-treated WT and Thy-1 KO mice are shown.
